# Assessing Structures and Solution Behaviors of Molecular and Ionic Cocrystals with a Common Bioactive Molecule: 2,4-Pyridinedicarboxylic Acid with Tranexamic Acid and Nicotinamide

**DOI:** 10.1021/acs.cgd.4c00525

**Published:** 2024-08-01

**Authors:** Charles Izuchukwu Ezekiel, Sanika Jadhav, Lewis L. Stevens, Leonard R. MacGillivray

**Affiliations:** † Department of Chemistry, University of Iowa, Iowa City, Iowa 52242, United States; ‡ Department of Pharmaceutical Sciences and Experimental Therapeutics, College of Pharmacy, University of Iowa, Iowa City, Iowa 52242, United States; § Department of Chimie, Université de Sherbrooke, Sherbrooke, Québec J1K 2R1, Canada

## Abstract

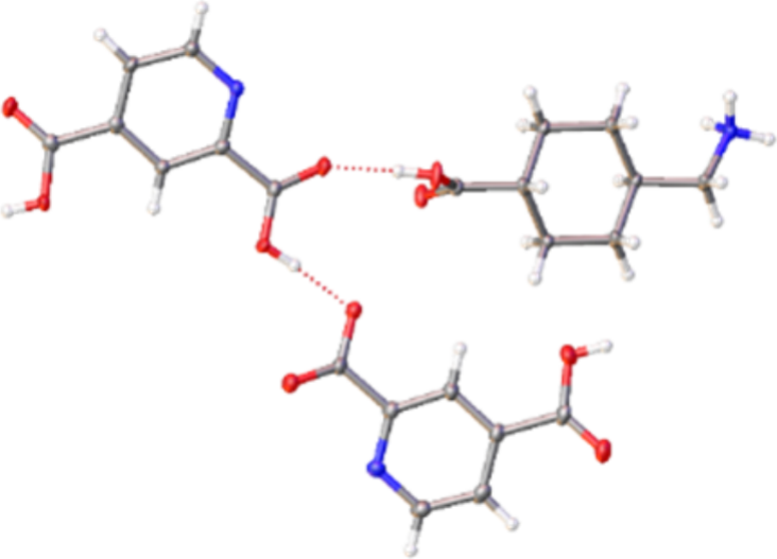

Cocrystals of 2,4-pyridinedicarboxylic acid (**PDA**) with either nicotinamide (**NTD**) or tranexamic acid (**TXA**) as (**PDA)·(NTD)** and **2(PDA)·(TXA)**, respectively, are reported, with the former being a molecular cocrystal and the latter being an ionic cocrystal. Single-crystal structure analyses showed that **PDA** and its coformers are sustained by neutral and ionic hydrogen bonds. Suspensions of **(PDA)·(NTD)** resulted in complete conversion to **PDA** monohydrate after 48 h, while **2(PDA)·(TXA)** was thermodynamically stable at a lower pH and showed a 2-fold increase in the **PDA** concentration, relative to pure **PDA** monohydrate under similar conditions. Thermal characterization of **2(PDA)·(TXA)** displayed a lower melting point and a lower heat of fusion, relative to the pure components. Powder dissolution studies were evaluated for **PDA**, **(PDA)·(NTD)**, and **2(PDA)·(TXA)** and the corresponding physical mixtures. The percent of **PDA** dissolved rapidly reached near 100% for most cases; however, for **2(PDA)·(TXA)**, complete dissolution was not achieved, and the amount of **PDA** dissolved decreased to 85% after 3 h. Instability of **2(PDA)·(TXA)** was likely a result of a high solution pH during dissolution, and our results confirm that the solution pH plays a key role in determining the solution behavior and phase stability of the cocrystals.

## Abstract 2

Molecular and ionic cocrystals of 2,4-pyridinedicarboxylic acid with either nicotinamide or tranexamic acid are reported, with solution data showing **PDA** dissolved to reach near 100% and the pH to play a major role in solution behavior and phase stability.

## Introduction

A critical objective in the development of solid pharmaceutical products is to enhance their physicochemical properties, especially solubility and permeability, while also preserving phase stability. Cocrystals have emerged as an avenue to improve the physicochemical properties of pharmaceutical solids such as solubility, dissolution, and stability, as well as bioavailability.^1−3^ Through crystal engineering, cocrystals have been developed to modify physicochemical and pharmacokinetic properties such as solubility and dissolution profiles, size and morphology, tableting and hydration stability, and bioavailability and permeability.^4^ Improving the physical properties of molecular solids through cocrystallization is not limited to pharmaceuticals, with properties of agrochemicals,^5^ explosives,^6^ and pigments^7^ also being enhanced.

Based on the ionization state of the molecular components, cocrystals can be generally classified as either “molecular” or “ionic”.^8^ Molecular cocrystals are composed of two or more components that exist in neutral forms (i.e., lack of charged functional groups), while ionic cocrystals are typically composed of a neutral and an ionic form. The components of an ionic cocrystal are often held together by charge-assisted hydrogen bonds.^9,10^

Cocrystallization strategies have recently been applied to develop solids based on the amphoteric bioactive molecule 2,4-pyridinedicarboxylic acid **(PDA)**. **PDA** is a potential inhibitor of human 2-oxoglutarate (2OG-oxygenases), which is implicated in a wide range of human diseases including cancer.^11,12^ From a crystal engineering standpoint, **PDA** possesses two hydrogen-bond donor carboxylic acid groups and a hydrogen-bond acceptor pyridyl group and can function as a zwitterion in the solid state. The integration of ionic components into cocrystals has been used to modulate physical properties (e.g., zwitterionic cocrystals) such as solubility and stability.^13,14^ Our group reported the zwitterionic pharmaceutical cocrystal **(PDA)**·**(APAP)** (**APAP** = acetaminophen), wherein **PDA** exists in a zwitterionic form. The binary cocrystal is bright orange-red in color, which contrasts the starting component solids that are colorless.^15^ While cocrystals of **PDA** have been reported with the pyridyl diacid in neutral and zwitterionic forms, we are unaware of an ionic cocrystal of **PDA**. Furthermore, there have been no reports of dissolution studies of cocrystals of **PDA**. The structure of **PDA** can essentially be considered a platform to allow for a broad range of cocrystal types to be investigated (i.e., molecular, zwitterionic, and ionic), which makes **PDA** unique to study structure–function relationships of cocrystals.

**Scheme 1:**
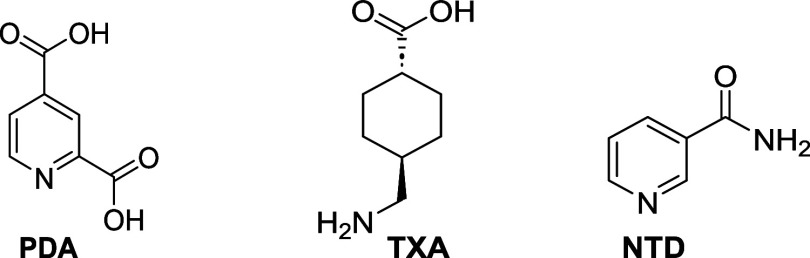
Structures of 2,4-Pyridinedicarboxylic Acid (**PDA**), Tranexamic Acid (**TXA**), and Nicotinamide (**NTD**)

Herein, we report structures of molecular and ionic cocrystals of **PDA** along with solution behaviors and phase stabilities (Scheme 1). Specifically, we show the cocrystallization of **PDA** with either nicotinamide (**NTD**) or tranexamic acid (**TXA**) to afford the molecular and ionic cocrystals **(PDA)·(NTD)** and **2(PDA)·(TXA)**, respectively. **TXA** is a pharmaceutical product and belongs to a class of drugs known as antifibrinolytics. **TXA** is used to treat extreme blood loss from surgery, trauma, and heavy menstrual bleeding,^16^ being on the World Health Organization (WHO)’s list of essential medicines.^17^**TXA** is also used in the management of prostate surgery, tonsillectomy, and cardiac surgery.^18−20^**NTD** is an amide of nicotinic acid (i.e., vitamin B3), with a current focus on the prevention of Type 1 diabetes mellitus. **NTD** is also a food additive for regulatory purposes. Crystal engineering approaches^21^ have formed multicomponent solids of **TXA**, with a search of the Cambridge Structural Database (CSD) revealing a total of 10 hits of organic and inorganic salts and cocrystals.^16,22−25^ As a pure form, **TXA** exists as a zwitterion in the solid state.^26^ None of the reported multicomponent solids of **TXA** exist, wherein either **TXA** or the coformer supports the generation of an ionic cocrystal. While there are a total of 428 hits for **NTD**,^27−31^ none describe a cocrystal of **NTD** with **PDA**. For **(PDA)·(NTD)** and **2(PDA)·(TXA)**, we show the components to assemble to form infinite arrays sustained by the combinations of neutral and/or ionic hydrogen bonds. Follow-on solution studies show that coformer selection and the solution pH significantly impact the resulting **PDA** concentrations. Cocrystal suspensions after 48 h reach a final pH of 2–3, and it was observed that **(PDA)·(NTD)** was unstable and transformed to **PDA** monohydrate. However, **2(PDA)·(TXA)** was stable and the concentration of **PDA** increased 2-fold, relative to **PDA** monohydrate solutions at a similar pH.

## Experimental Section

All reagents and solvents were purchased from commercial sources and used as received, unless stated otherwise. **PDA** and **TXA** were purchased from Ambeed Inc. and Oakwood Chemical, respectively. **NTD** was purchased from Aldrich.^©^ Ethanol, dimethyl sulfoxide, and diethyl ether were purchased from Millipore-Sigma. Cocrystal syntheses were conducted in screw-capped scintillation vials.

Equimolar **PDA** (15.0 mg, 0.09 mmol) and **NTD** (11.0 mg, 0.09 mmol) were dissolved separately in ethanol/ether (1:1 vol) with minimal heat, and the solutions were mixed in a 10 mL scintillation vial and allowed to slowly evaporate at room temperature. Equimolar **PDA** (21.8 mg, 0.13 mmol) and **TXA** (20.5 mg, 0.13 mmol) were dissolved separately in ethanol with minimal heat, and the solutions were mixed in a 10 mL scintillation vial and allowed to slowly evaporate at room temperature. Single crystals suitable for X-ray diffraction analysis formed in both cases after a period of approximately 48 h.

### Powder X-ray Diffraction (PXRD)

Samples for PXRD analyses were ground by using a mortar and pestle to generate a uniform powder, which was then deposited on a KS Analytics zero background holder and analyzed with a Bruker D8 Advanced PXRD diffractometer. Data were collected over the range of 5–40° 2θ using a 1.5 s step with synchronous rotation of the sample holder. Single-crystal compositions were representative of bulk materials by matching the experimental PXRD patterns and single-crystal X-ray diffraction data. Stoichiometries of materials were determined by SCXRD and ^1^H NMR spectroscopy.

### Single-Crystal X-ray Diffraction (SCXRD)

SCXRD experiments were performed using a Bruker D8 Quest diffractometer equipped with an Oxford Cryosystem. Absorption correction was applied with the SADABS multiscan method within APEX3.^32^ Individual single crystals suitable for the analyses were secured on magnetic mounts with Paratone oil and mounted on the instrument. All measurements were carried out at 100 K using either Cu Kα (λ = 1.54178 Å) radiation for **(PDA)·(NTD)** or Mo Kα (λ = 0.71073 Å) radiation for **2(PDA)·(TXA)**. Olex2^33^ was used to solve the crystal structures using direct methods and refined with SHELXS and SHELXL packages.^34^

### Preparation of Cocrystals for Solution Behaviors

Mechanochemical syntheses of the cocrystals were performed by using a FTS-1000 shaker mill. Liquid-assisted grinding (LAG) was employed to mill an equimolar mixture of the individual components with 10 μL of ethanol/ether (1:1) for **(PDA)·(NTD)** and ethanol for **2(PDA)·(TXA)** in stainless steel jars (5 mL) with steel ball bearings (5 mm) at 20 Hz for 30 min. The cocrystals formed quantitatively, as confirmed by experimental PXRD and single-crystal data.

### Differential Scanning Calorimetry (DSC)

Thermal analyses were performed using a DSC Q20 differential scanning calorimeter (TA Instruments) with a constant nitrogen purge flow of 40 mL/min. Approximately 1–5 mg of the sample was weighed in an aluminum pan and crimped with a lid. Samples were heated from 25 to 350 °C at a heating rate of 5 and 20 °C/min. Care was taken to maintain a uniform layer of the sample at the bottom of the pan for efficient heat flow measurement. The DSC was calibrated for temperature and cell constant using an indium standard, and an empty sealed pan was used as a reference standard. All thermograms were analyzed using Universal TA Analysis software.

### Solution Studies and Phase Stabilities

Cocrystal suspensions were prepared, and **PDA** concentrations were measured after 48 h. All experiments were performed at 25 °C in 20 mL screw-capped vials with excess powdered solids (200–250 mg for each cocrystal) added to 1 mL of water or phosphate buffer (pH 6.8) at concentrations of 100 or 500 mM. All suspensions were shaken at 150 rpm using a rotary shaker (Thermo Scientific, MA). After 48 h, each sample was centrifuged for 5 min at 3000 rcf, and **PDA** concentrations were analyzed using high-performance liquid chromatography (HPLC). The solid residue was dried in an oven for 4 h at 60 °C and analyzed using PXRD. The solution pH was recorded at the beginning and end of each experiment using pH paper (Fisherbrand). Note that the solubility of **TXA** was determined gravimetrically, owing to the absence of a chromophore.

### Powder Dissolution Studies

Solid samples of **PDA**, **(PDA)·(NTD)**, and **2(PDA)·(TXA)** and physical mixtures (PMs) were initially sieved using a sonic sifter (Allen-Bradley Sonic Sifter Model L3P, WI). Fractions captured between mesh sizes 150–250 μm were used for the dissolution studies. Powder dissolution studies were conducted in a 20 mL screw-capped vial at 150 rpm using a rotary shaker (Thermo Scientific, MA) in phosphate buffer (pH 6.8, 500 mM) at 25 °C. Approximately 100 mg (*n* = 3) was added to 3 mL of dissolution media and 0.5 mL samples were taken at various time intervals. After each sampling, the same volume was replaced with fresh media. All samples were then passed through 0.22 μm PES membrane filters and diluted appropriately with a 1:1 ratio of the mobile phase prior to HPLC analysis. The pH of each sample was recorded before and at the end of the experiment, and depending on the quantity present, the solid residue remaining at the end of dissolution was analyzed using PXRD.

## Results and Discussion

### Crystal Structure of **(PDA)·(NTD)**

**Figure 1:**
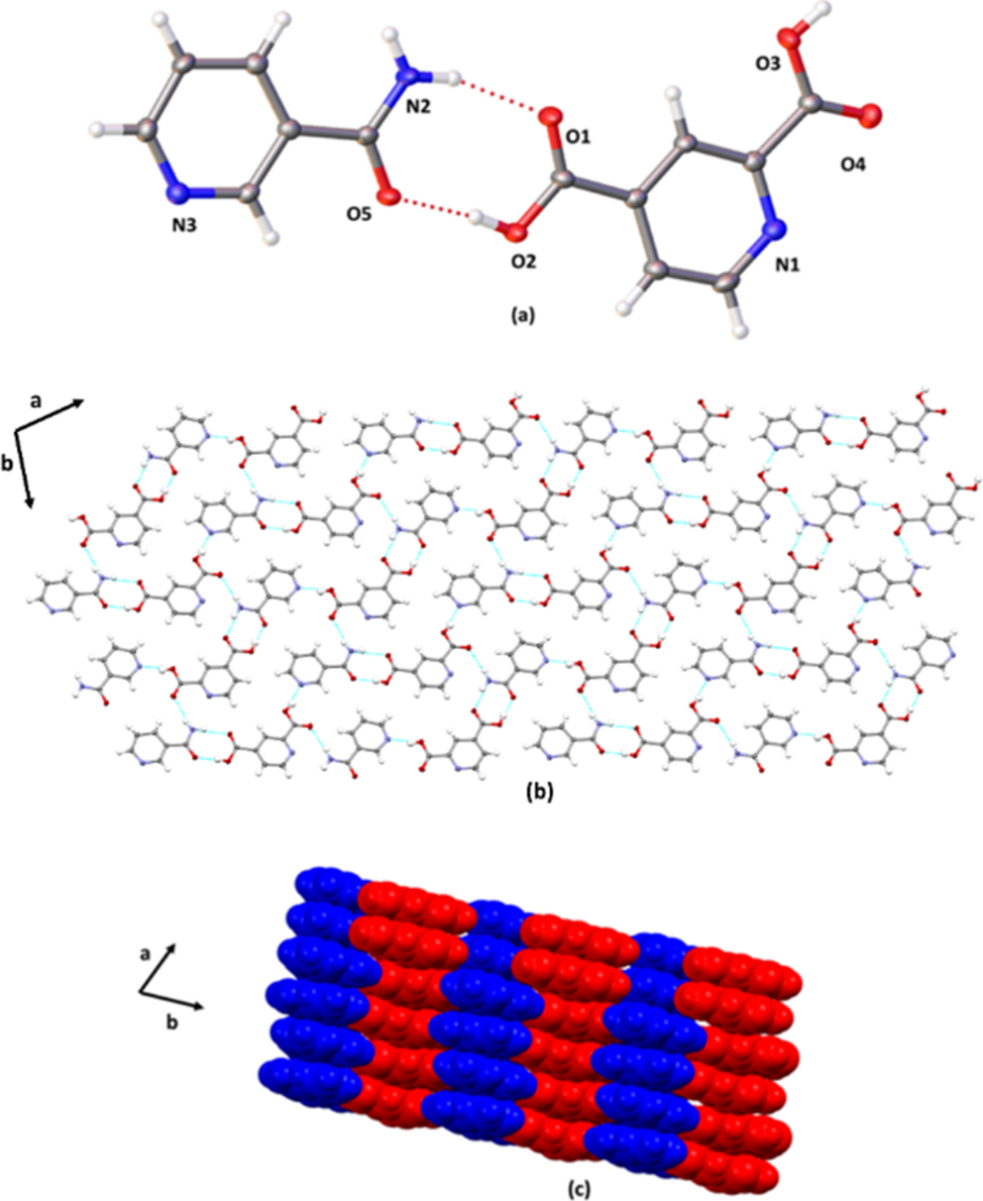
X-ray structure of **(PDA)·(NTD)**: (a) asymmetric unit, (b) 2D layer, and (c) layer packing (space filling).

The components of **(PDA)·(NTD)** crystallize in the monoclinic space group *Pc*, with the asymmetric unit consisting of one full molecule each of **PDA** and **NTD** (Figure 1). The molecules assemble by a combination of acid-amide (O2···O5 2.574(2) Å and ∠N–H···O 173°; N2···O1 2.877(3) Å and ∠N–H···O 162°) and acid-pyridine (O3···N3 2.592(3) Å and ∠O–H···N 165°) supramolecular heterosynthons that form a one-dimensional (1D) array (Figure 1a). **PDA**, thus, exists as a neutral form (C=O(1) 1.218(3), C–O(2) 1.315(3), C=O(4) 1.223(3), and C–O(3) 1.308(3) Å). Adjacent arrays are linked by amide-pyridine heterosynthons (N1···N2 3.065(3) Å) to generate a two-dimensional (2D) layered structure within the *ab*-plane (Figure 1b). The layers stack along the crystallographic *c*-axis by face-to-face π–π interactions involving the pyridine rings (3.667(4) Å) (Figure 1c). Nearest-neighbor **NTD** molecules interact through C–H···O hydrogen bonds (3.356(3) Å).

### Crystal Structure of **2(PDA)·(TXA)**

**Figure 2:**
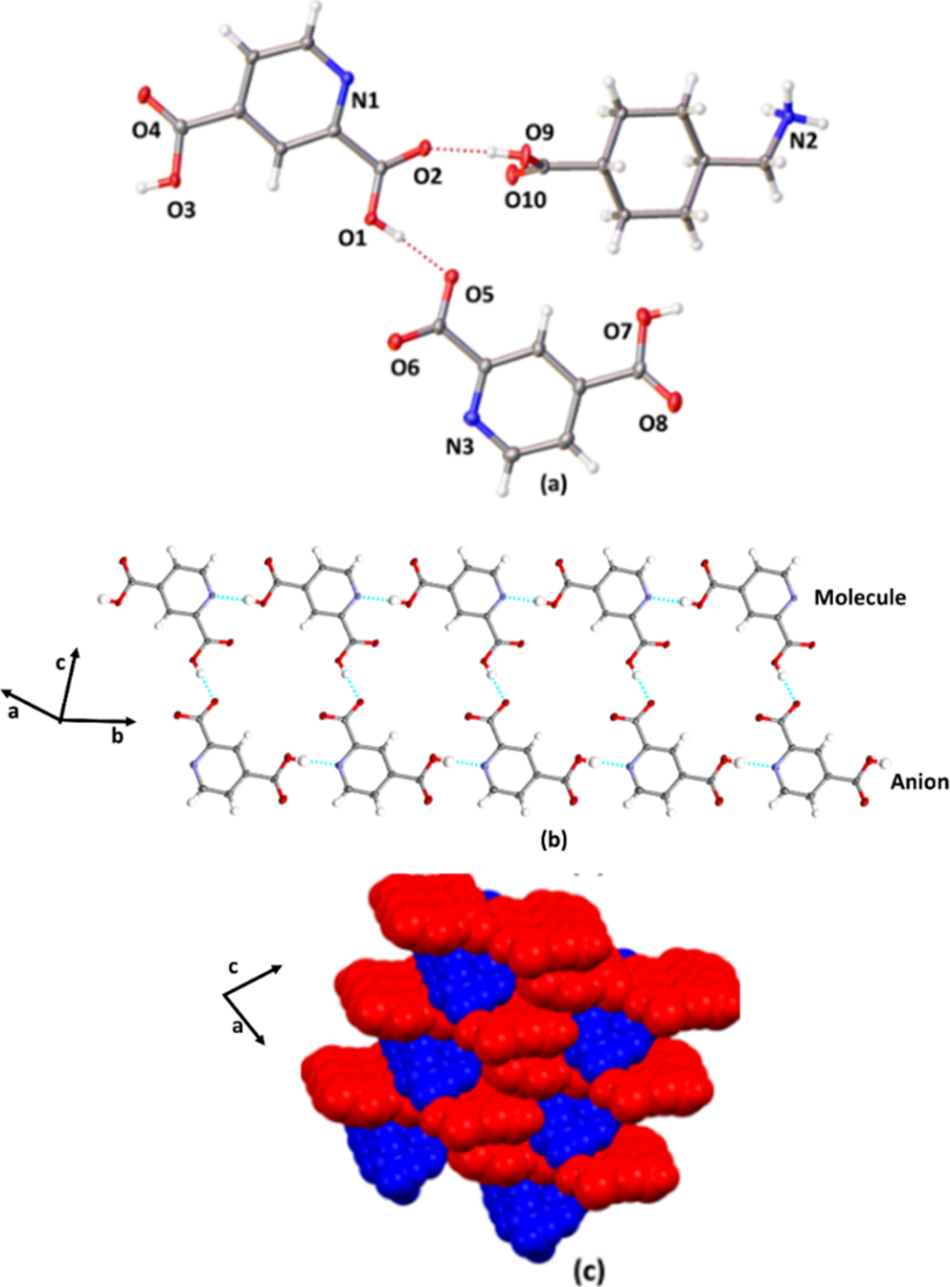
X-ray structure **2(PDA)·(TXA)**: (a) asymmetric unit, (b) zipper assembly, and (c) bricklike packing (space filling).

The components of **2(PDA)·(TXA)** crystallize in the chiral triclinic space group *P*1, with the asymmetric unit consisting of cationic **TXA**, anionic **PDA**, and neutral **PDA** (Figure 2). The components thus form an ionic cocrystal involving a **TXA**^**+**^**PDA**^–^ ion pair (Figure 2a). Proton transfer involves the ortho acid group of **PDA** and the amino group of **TXA** (**PDA** anion: C=O(2) 1.239(5), C–O(1) 1.272(5); C=O(4) 1.213(5), C–O(3) 1.315(5); **PDA** neutral: C=O(6) 1.226(5), C–O(5) 1.292(4), C=O(8) 1.195, C–O(7) 1.318(5); **TXA** cation C=O(10) 1.217(5), C–O(9) 1.324(4) Å). The components self-assemble to form a 1D zipper-like topology sustained by a combination of O–H···O^–^ (O1···O5 2.483(3) Å; ∠O–H···O^–^ 166°), O–H···O (O9···O2 2.534(4) Å; ∠O–H···O 165°), O–H(molecule)···N (O3···N1 2.743(5) ∠O–H···N 174°), and O–H(anion)···N (2.742(5) ∠O–H···N 175°) hydrogen bonds involving **PDA** anions and molecules (Figure 2b). Adjacent zippers are linked by the **TXA** cations through a combination of O–H···O and N^+^-H···O hydrogen bonds (O9···O2 2.534(4) Å; ∠O–H···O 165°, and N2···O6 2.782(4) Å; N^+^–H···O 160°). The zippers pack offset and interdigitate to form a bricklike framework along the *a*-axis (Figure 2c).

### Thermal Characterization of PDA Cocrystals

Thermal analyses of **PDA**, **NTD**, **TXA**, and cocrystals as well as physical mixtures were performed to assess the strength of lattice interactions. The DSC thermograms of **PDA**, **TXA**, and **NTD** showed single endotherms at 247.32 °C (Δ*H*_f_ = 161.1 J/g), 303.20 °C (Δ*H*_f_ = 474.4 J/g), and 128.66 °C (Δ*H*_f_ = 183.1 J/g), respectively.

**Figure 3:**
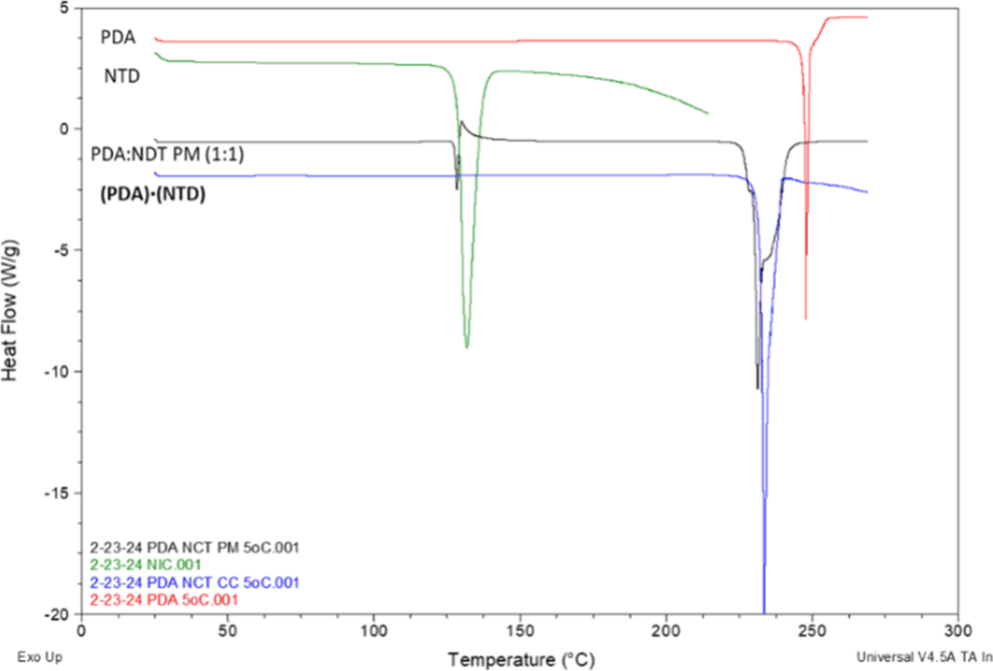
Overlay of DSC thermograms of **PDA**, **NTD**, PM, and **(PDA)·(NTD)**.

For **(PDA)·(NTD)**, there was a single melt at 232.63 °C (Δ*H*_f_ = 633.3 J/g), which is intermediate to the pure components (Figure 3). For a physical mixture of **PDA** and **NTD**, there was a small endotherm at 127.62 °C (Δ*H*_f_ = 27 J/g), followed by a small exotherm at 129.89 °C (Δ*H*_f_ = 41.96 J/g). At higher temperatures, a broad endotherm with multiple features was observed in the range of 223.05–246.66 °C. We suggest that heating the physical mixture initially results in the formation of a eutectic that is followed by recrystallization that presumably leads to the cocrystal.

**Figure 4:**
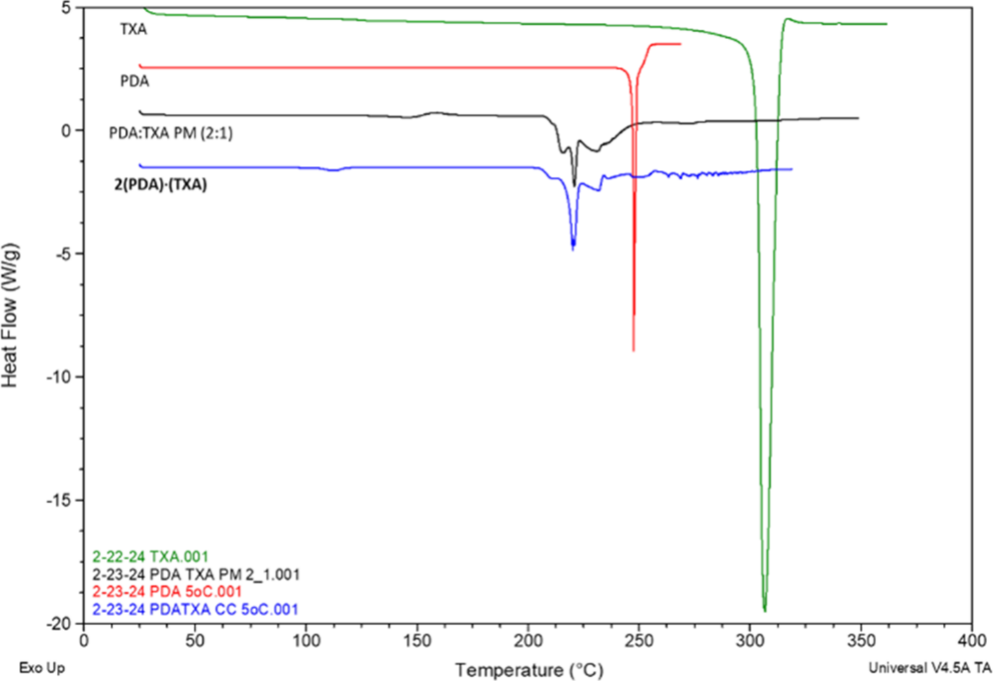
Overlay of DSC thermograms of **PDA**, **TXA**, PM, and **2(PDA)·(TXA)**.

For **2(PDA)·(TXA)**, there was a small endotherm at 105.83 °C (Δ*H*_f_ = 10.81 J/g) and then a broad endotherm with shoulders at approximately 218.07 °C (Δ*H*_f_ = 108.9 J/g) (Figure 4). The endotherm near 105 °C may be ascribed to a loss of adsorbed water, as ionic-based materials are often hygroscopic.^35^ The lower melting temperature for **2(PDA)·(TXA)** suggests that the cocrystallization resulted in weaker overall lattice interactions versus the pure components. Using a database of 727 cocrystals, Perlovich categorized the melting behavior of cocrystals relative to individual components.^36^ For 2:1 (or 1:2) cocrystals, the cocrystal having the lowest melting temperature occurred only 23.5% of the time. A physical mixture of **PDA** and **TXA** showed a small endotherm followed by an exotherm at 132.64 °C (Δ*H*_f_ = 21.97 J/g), which was followed by a broad endotherm that exhibits multiple features in the range of 204.95–255.74 °C. The initial peak, similar to the PM of **PDA** and **NTD**, suggests eutectic formation followed by recrystallization to form the cocrystal.

### Solution Studies

**Figure 5:**
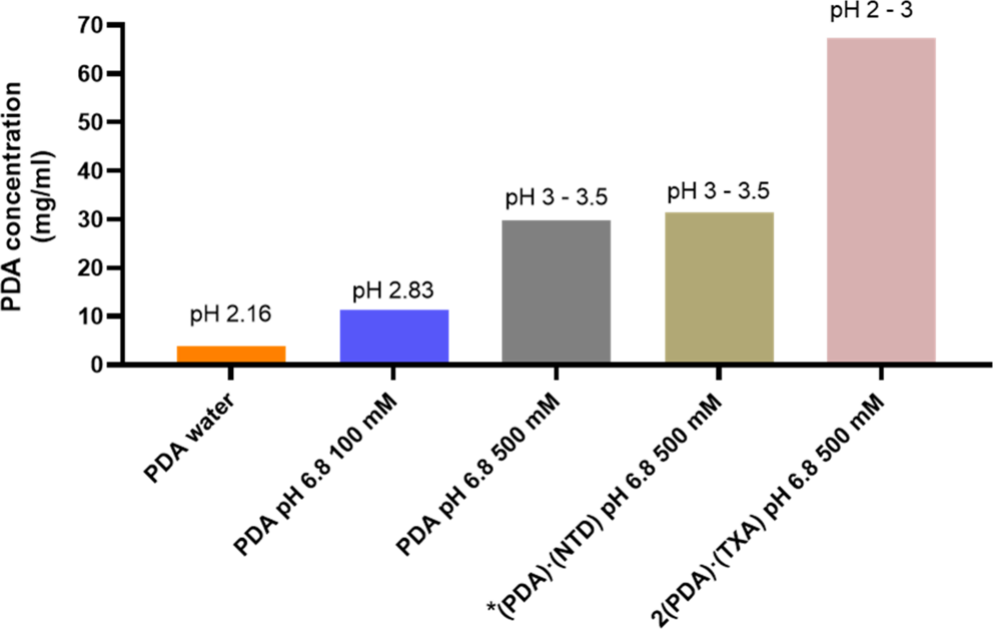
Comparison of **PDA** concentrations for different solid forms after 48 h at 25 °C at specific buffer conditions. * **(PDA)·(NTD)** was unstable and transformed to **PDA** monohydrate.

Two p*K*_a_ values (both acidic) are reported for **PDA**, 2.17 and 5.17. We found that, initially, anhydrous **PDA** underwent a solution-mediated phase transformation to **PDA** monohydrate after 48 h (Figure S2). Further, the solubility of **PDA** monohydrate increased from 3.8 to 11.2 mg/mL as the solution pH increased from 2.16 to 2.83, a result of the higher percent ionization of a carboxylic acid (Table 1 and Figure 5). At the higher pH range of 3.0–3.5, the solubility of **PDA** monohydrate increased further to 29.8 mg/mL, which can be attributed to continued ionization. Interestingly, at high buffer concentrations (500 mM), anhydrous **PDA** converted to a mixture of **PDA** monohydrate and an unknown solid phase. Despite using the high buffer concentration, adding sufficient **PDA** to reach saturation resulted in a low solution pH; thus, it is important to measure the pH at the start and end of the solubility studies, particularly for ionizable molecules. **NTD** and **TXA** showed high solubilities of 415.6 and 180.8 mg/mL, respectively, and no phase conversions over a period of 48 h (Figures S3 and S4).

**Table 1:** Total **PDA** Solution Concentrations and Phase Stabilities for Different Solid Forms

solid form	media	final pH	concentration after 48 h (mg/mL)	solid phase at the bottom of the vial after 48 h
PDA	phosphate buffer (500 mM) pH 6.8	∼3–3.5	29.8	PDA monohydrate and unknown phase
PDA	phosphate buffer (100 mM) pH 6.8	2.83	11.2 ± 0.3	PDA monohydrate
PDA	water	2.16	3.8 ± 0.1	PDA monohydrate
(PDA)·(NTD)	phosphate buffer (500 mM) pH 6.8	∼3–3.5	31.4 ± 1.0 (PDA)	PDA monohydrate
2(PDA)·(TXA)	phosphate buffer (500 mM) pH 6.8	∼2–3	67.3 ± 1.5 (PDA)	2(PDA)·(TXA)

For the cocrystals, **(PDA)·(NTD)** was unstable in solution and converted to **PDA** monohydrate after 48 h. For **2(PDA)·(TXA)**, no phase conversion was observed after 48 h (Figures S5 and S6) and the total concentration of **PDA** in solution increased to 67.3 mg/mL, a 2-fold increase relative to **PDA** monohydrate. Key factors that may contribute to the increased concentration of **PDA** from **2(PDA)·(TXA)** include the high solubility of **TXA**, the amine ionization of **TXA** at pH 2–3, and the reduction in the strength of lattice interactions for **2(PDA)·(TXA)**, as illustrated by a lower melting point and heat of fusion. We could not determine the aqueous cocrystal solubility for either cocrystal as the instability of **(PDA)·(NTD)** resulted in **PDA** monohydrate and not a eutectic, and the **TXA** concentration for **2(PDA)·(TXA)** could not be determined owing to a lack of a chromophore. Based on instability, it is possible that **(PDA)·(NTD)** has a higher cocrystal solubility than **2(PDA)·(TXA)** but additional studies are needed.

### Powder Dissolution Profiles

As determined from the solution studies, saturating a solution with **PDA** lowers the pH significantly, even if buffered. For dissolution studies, we selected the high-concentration phosphate buffer (pH 6.8, 500 mM) to ensure that all dissolution profiles were evaluated at a consistent pH. Measurements of the pH before and after dissolution showed only minor differences. At pH 6.8, **PDA** is fully ionized and differences in **PDA** dissolution may be compared more directly. Moreover, the dissolution profiles of PMs were compared with those of the corresponding cocrystals to assess how **PDA** cocrystallization impacts dissolution.

**Figure 6:**
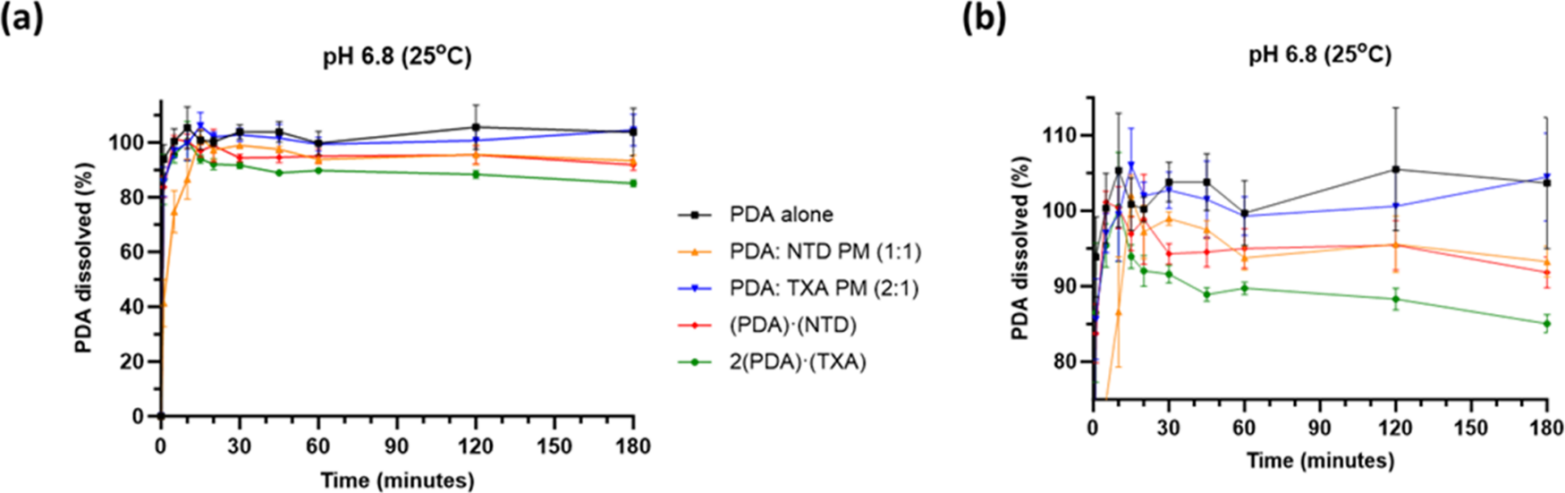
Full (a) dissolution profiles of **PDA**, PMs, **(PDA)·(NTD)**, and **2(PDA)·(TXA)** in phosphate buffer at pH 6.8 and (b) zoomed versions highlighting the decrease in the percent of **PDA** dissolved from **2(PDA)·(TXA)**.

Dissolved **PDA** from all solids quickly increased to above 80% within 5 min owing to a high solubility of **PDA** (Figure 6). **PDA** and **PDA:TXA** (2:1) PMs fully dissolved, and solutions remained clear with no signs of precipitation after 3 h. The percent of **PDA** dissolved from **PDA:NTD** (1:1) PM reached 100% in 15 min and slowly decreased, but without evident precipitation. For **2(PDA)·(TXA)**, **PDA** dissolved approached 100% after 10 min but the complete powder mass never fully dissolved, and over time, the percent of **PDA** dissolved steadily decreased to 85.0% (Table 2), perhaps as a result of **2(PDA)·(TXA)** recrystallization. For (**PDA)·(NTD)**, slight crystallization of likely **PDA** monohydrate was observed specifically at the air–liquid interface but did not continue; thus, the percent of **PDA** dissolved was less impacted and decreased to 91.9% after 3 h. In neither case was there sufficient solid residue available to fully establish phase identity by PXRD.

**Table 2:** Dissolution Profiles for **PDA**, PMs, and Cocrystals in Phosphate Buffer at pH 6.8 (500 mM) at 25 °C

solid form	initial pH	final pH	% PDA dissolved after 180 min (mean ± SD)	solid residue after the end of the experiment	*p*-value^a^
PDA	6.8	6.20	103.69 ± 8.72	no residue; entire powder dissolved	ns
PDA:NTD PM (1:1)	6.8	6.34	93.28 ± 2.06	no residue; entire powder dissolved	ns
PDA:TXA PM (2:1)	6.8	6.28	104.47 ± 5.79	no residue; entire powder dissolved	0.0046 (w.r.t. 2(PDA)·(TXA))
(PDA)·(NTD)	6.8	6.73	91.86 ± 2.03	entire mass dissolved; precipitation started after 60 min at the air–liquid interface	ns
2(PDA)·(TXA)	6.8	6.64	85.04 ± 1.20	entire mass did not dissolve	0.0061 (w.r.t. PDA)

a Note: the *p*-value was calculated using Turkey’s multiple comparison test by comparing the mean of the % of PDA dissolved at 180 min; ns: statistically nonsignificant.

Statistical analyses were performed for the percent of **PDA** dissolved at 180 min across all materials using one-way analysis of variance (ANOVA) followed by Tukey’s multiple comparison test (see Table 2 for *p*-values). The percent **PDA** dissolved involving **2(PDA)·(TXA)** was significantly *lower*, relative to those of **PDA** and PMs of **PDA:TXA**. Interestingly, the observation is despite the improved **PDA** concentration achieved by **2(PDA)·(TXA)** at pH 2–3 and points to a crucial role of the solution pH. At the higher pH of the dissolution studies, all **TXA** in the solution is zwitterionic (predicted p*K*_a_’s 4.56 (acidic), 10.22 (basic)) and net neutral, which may reduce **TXA** solubility (and likely cocrystal solubility), relative to positively charged **TXA** at pH 2–3. Thus, for all solution studies, it is important to record the pH at the start and end of the experiment. Overall, the cocrystallization of **PDA** with **NTD** and **TXA** had a modest impact on the dissolution of **PDA** at pH 6.6, but through different mechanisms.^37,38^

## Conclusions

Molecular and ionic cocrystals of **PDA** were generated by involving **NTD** and **TXA**. The solids were sustained by neutral and charged-assisted hydrogen bonds. Solution studies demonstrated the ionic cocrystal, **2(PDA)·(TXA)**, to be stable for 48 h at a lower pH and the **PDA** concentration achieved improved 2-fold relative to **PDA** monohydrate at similar conditions. The observation could be attributed to a combination of the high water solubility of **TXA**, the positive ionization of the amine, and a lower melting point of the cocrystal. Powder dissolution profiles for all materials reached complete dissolution except for **2(PDA)·(TXA)**. From a development perspective, **PDA** cocrystallization with **NTD** or **TXA** yielded two distinct results. Highly soluble **NTD** increased the solubility of **(PDA)·(NTD)** but at the risk of **PDA** precipitation, and after complete dissolution of **(PDA)·(NTD)**, a slight precipitate was ascribed to **PDA** monohydrate. The solution pH played a critical role as **PDA** and **TXA** are both ionizable and impacted the cocrystal performance uniquely at different pH values. **2(PDA)·(TXA)** was stable at pH 2–3 but during dissolution at pH 6.6, it lowered the percent of **PDA** dissolved, relative to pure **PDA** and **PDA:TXA** PMs. Thus, molecular or ionic cocrystals of water-soluble drugs could be explored to manage pharmaceutically relevant properties, but coformers need to be carefully considered based on solubility and potential ionization. Future studies are warranted to understand the overall performance of cocrystals and their roles in formulation.

## Supporting Information Available

Details of chemicals used, SCXRD and PXRD data, ^1^H NMR data, and thermal analyses (PDF)
