# Supplementary material for: Assessing Structures and Solution Behaviors of Molecular and Ionic Cocrystals with a Common Bioactive Molecule: 2,4-Pyridinedicarboxylic Acid with Tranexamic Acid and Nicotinamide
Source: Cryst Growth Des. 2024 Aug 1;24(16):6618–24. doi: 10.1021/acs.cgd.4c00525 (PMC11342296; doi:10.1021/acs.cgd.4c00525)
Supplement: Supplementary file 1 — cg4c00525_si_001.pdf [file cg4c00525_si_001.pdf]

Supporting Information

Assessing Structures and Solution Behaviors of Molecular and Ionic Cocrystals with a Common Bioactive Molecule: 2,4-Pyridinedicarboxylic Acid with Tranexamic Acid and Nicotinamide

Charles Izuchukwu Ezekiel[1], Sanika Jadhav[2], Lewis L. Stevens*[2] and Leonard R. MacGillivray[*3]

[1]Department of Chemistry, University of Iowa, Iowa City, IA 52242 USA

[2]Department of Pharmaceutical Sciences and Experimental Therapeutics, College of Pharmacy, University of Iowa, Iowa City, IA 52242, USA

[3] Department de chimie, Université de Sherbrooke, Sherbrooke, QC, J1K 2R1, Canada

Supplementary Information

S1. Methods

S2. Bond Metrics and Single Crystal X-ray Diffraction Data

S3. Powder X-ray Diffraction (PXRD) Data

S4. Nuclear Magnetic Resonance (NMR) Spectroscopy Data

S5. UV-Vis and HPLC Data

S6. References

S1. Methods

Synthesis and X-ray Analysis. Each cocrystal was prepared by mixing equimolar amounts of components. The syntheses were performed in screw-cap scientillation vials. Equimolar amounts of **PDA** and each coformer were dissolved separately in either EtOH or 1:1 EtOH and ether by applying minimal heat, the solutions were mixed and allowed to slowly evaporate at room temperature. Crystals suitable for diffraction analysis were achieved after periods on the order 48 hours.

X-ray Crystallography. Single crystal X-ray diffraction experiments were performed using Bruker D8 Quest diffractometer with an Oxford Cryosystem. Absorption correction was applied with the SADABS multi-scan method within APEX3.[1] The single crystals suitable for the diffraction analyzes were secured on the magnetic mounts with Paratone oil and mounted on the instrument. The measurements were carried using Mo Kα (λ = 0.71073 Å) radiation for **2(PDA)·(TXA)** and Cu Kα (λ = 1.54178 Å) radiation for **(PDA).(NTD).** Olex2[2] was used to solve the crystal structures using direct methods and refined with SHELXS and SHELXL packages.[3]

Powder X-ray diffraction. Powder X-ray diffraction (PXRD) data were collected on a Bruker D8 Advance X-ray diffractometer using CuKα1 radiation (λ = 1.5418 Å) in the range 5–40° (scan type: coupled TwoTheta/Theta; scan mode: continuous PSD fast; step size: 0.019°) (40 kV and 30 mA).

NMR spectroscopy. Proton nuclear magnetic resonance (^{1}H NMR) spectra was recorded at room temperature on a Bruker® DRX-400 spectrometer at 400 MHz (Instrument parameters: field strength : 9.2 T; RF-Console: DRX 3-channel; magnet: shielded superconducting; probe: nature, 5.0 mm BBO-^{1}H/^{15}N-^{31}P; type, double-resonance; temperature range: 100-180 °C).

Differential Scanning Calorimetry (DSC). Thermal analysis on all solids was done using a DSC Q20 (TA instruments, USA) with a constant nitrogen purge flow of 40 mL/min. Approximately 1-5 mg of sample was weighed in an aluminum pan and crimped with a lid. Samples were heated from 25°C to 350°C at a heating rate of 5°C/min and 20°C/min. Care was taken to maintain a uniform layer of sample at the bottom of the pan for efficient heat flow measurement. The DSC was calibrated for temperature and cell constant using an indium standard, and an empty sealed pan was used as a reference standard. All thermograms were analyzed using the Universal TA analysis software.

High Performance Liquid Chromatography (HPLC)

A HPLC method was developed to obtain well-formed and resolved peaks of **PDA** and each coformer. Reverse-phase HPLC was performed using an Agilent 1100 HPLC (CA, USA) equipped with a DAD detector with a nominal wavelength range from 190 – 950 nm. An Agilent Eclipse XDB C18 column, with a particle size of 5 µm (4.6 x 150 mm), was used as the stationary phase. Methanol and phosphate buffer (pH 6.8, 10mM) in a 30:70 ratio respectively was used as the mobile phase with a flow rate of 0.5 ml/min and a sample injection volume of 10 µl. **PDA** and **NTD** were detected at 276 and 262 nm respectively. Each wavelength was determined from UV spectra from a microplate reader (Molecular Devices SpectraMax® M5, CA, USA) (**Figure S4**). Standard stock solutions were prepared by transferring 10 mg of accurately weighed material into a 10-mL volumetric flask and dissolving in methanol (1 mg/mL). Serial dilutions were prepared for standard analysis in triplicate and generation of a calibration curve. The calibration curve displayed a linear

working range of 7.81 - 500 µg/ml with an r^2= 0.999, as reported in **Figure S2**. The retention time for **PDA** was 2.5 minutes which was well separated compared to **NTD** retention time of 3.6 minutes (**Figure S3**). Owing to absence of a chromophore in **TXA**, its concentration could not be measured by spectrophotometry.

S2. Bond Metrics and Single Crystal X-ray Diffraction Data

Table S1. Hydrogen bonds in (PDA)·(NTD).

D-H···A	d(D···A), Å	∠ D-H···A, deg
O(2)-H(2)···O(5)	2.574 (2)	173
N(2)-H(2A)···O(1)	2.877 (3)	162
N(2)-H(2B)···O(4)i	3.066 (3)	168
O(3)-H(3)···N(3)^{ii}	2.592 (3)	165
C(4)-H(4)···O(1)^{iii}	3.143 (3)	138
C(10)-H(10)···O(5)	2.752 (3)	101
C(12)-H(12)···O(5)^{iv}	3.407 (3)	161
C(30)-H(13)···O(4)^{iii}	3.300 (3)	167

Symmetry codes: (i) *x*, 1+*y*, *z*; (ii) -1+*x*, -*y*, -1/2+*z*; (iii) -1+*x*, -1-*y*, -1/2+*z*; (iv) 1+*x*, 1-*y*, ½+*z*

Table S2. Hydrogen bonds in 2(PDA)·(TXA)

D-H···A	d(D···A), Å	∠ D-H···A, deg
O(1)-H(1)···O(5)	2.483 (3)	166
N(2)-H(2A)···O(10)^{ii}	2.831 (4)	161
N(2)-H(2B)···O(9)^{iii}	2.896 (4)	163
N(2)-H(2C)···O(1)^{iv}	2.869 (4)	107
N(2)-H(2C)···O(6)^{iv}	2.782 (4)	160
O(3)-H(3)···O(2)^{i}	2.809 (5)	101
O(3)-H(3)···N(1)^{i}	2.742 (4)	174
O(7)-H(7)···N(3)^{ii}	2.742 (4)	175
O(9)-H(9)···O(2)	2.534 (4)	165
C(22)-H(22B)···O(4)^{v}	3.260 (5)	125

Symmetry codes: (i) *x*, -1+*y*, z (ii) *x*, 1+*y*, *z*; (iii) 1+*x*, 1+*y*, z (iv) 1+*x*,2+*y*, *z* (v) 1+*x*, 2+*y*, 1+*z*

Table S3. Selected Bond Distances for (PDA)·(NTD)

O(1)	C(1)	1.218 (3)
O(2)	C(1)	1.315 (3)
O(3)	C(7)	1.308 (3)
O(4)	C(7)	1.223 (3)

Table S4. Selected Bond Distances for 2(PDA)·(TXA)

O(1)	C(13)	1.272 (5)
O(2)	C(13)	1.239 (5)
O(3)	C(14)	1.315 (5)
O(4)	C(14)	1.213 (5)
O(5)	C(7)	1.292 (4)
O(6)	C(7)	1.226 (5)
O(7)	C(6)	1.318 (5)
O(8)	C(6)	1.199 (5)
O(9)	C(21)	1.324 (4)
O(10)	C(21)	1.217 (5)

Table S5. Crystallographic data and structure refinement parameters **(PDA)·(NTD) and 2(PDA)·(TXA)**

Identification code	**(PDA)·(NTD)**	**2(PDA)·(TXA)**
CCDC deposition number	2347033	2347034
Empirical formula	$C_{13}H_{11}N_3O_5$	$C_{22}H_{25}N_3O_{10}$
Formular weight g/mol	289.25	491.45
Temperature/K	100.0	100.0
Crystal system	monoclinic	triclinic
Space group	Pc	P1
a/Å	3.667(10)	7.420(3)
b/Å	14.168(3)	7.648(2)
c/Å	11.874(2)	10.359(4)
α/°	90	91.057(18)
β/°	95.263(15)	93.722(15)
**γ/°**	90	112.790(10)
Volume/$Å^3$	616.84(2)	540.20(3)
Z	2	1
ρ_{calc} (g/cm^3)	1.557	1.511
μ/mm^{-1}	1.042	0.121
F(000)	300.0	258.0
Crystal size/mm^3	0.045x0.093x0.191	0.148×0.221×0.228
Goodness-of-fit on F^2	1.084	1.046

S3. Powder X-ray Diffraction (PXRD) Data

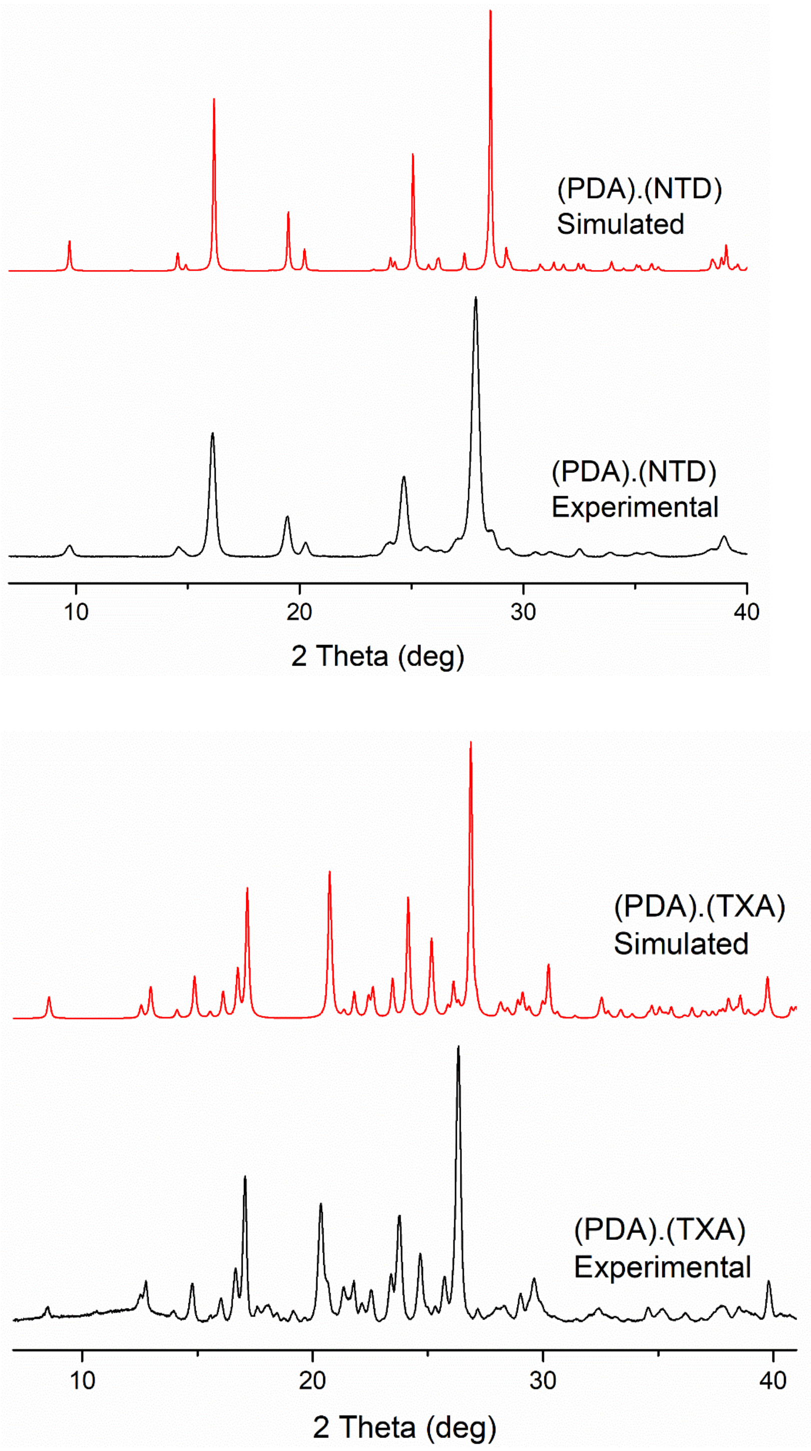

Figure S1. Experimental and simulated PXRD patterns of **(PDA)·(NTD)** and **2(PDA)·(TXA).**

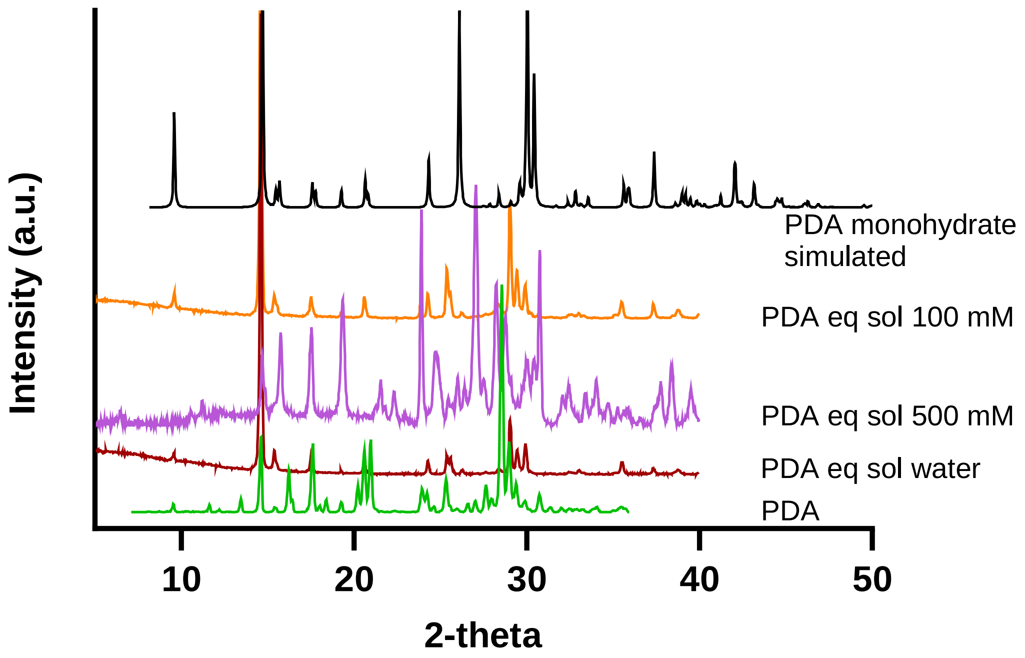

Figure S2. Overlays of powder X-ray diffraction patterns of **PDA**, **PDA** monohydrate and solid residues obtained after equilibrium solubility studies.

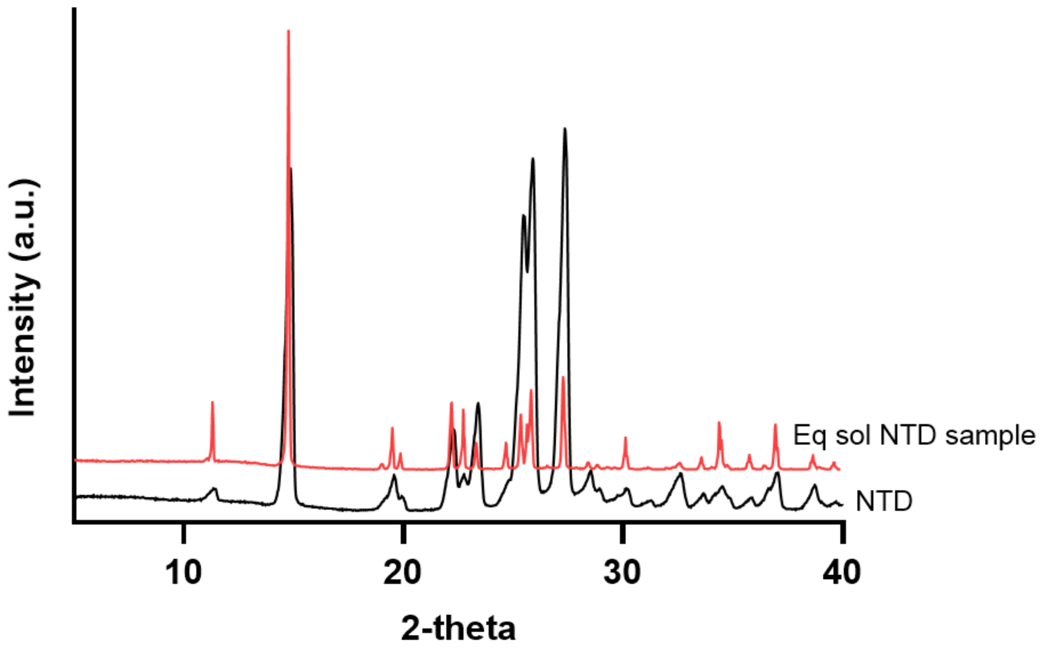

Figure S3. Overlay of powder X-ray diffraction patterns of **NTD** and solid residue obtained after equilibrium solubility studies of **NTD**.

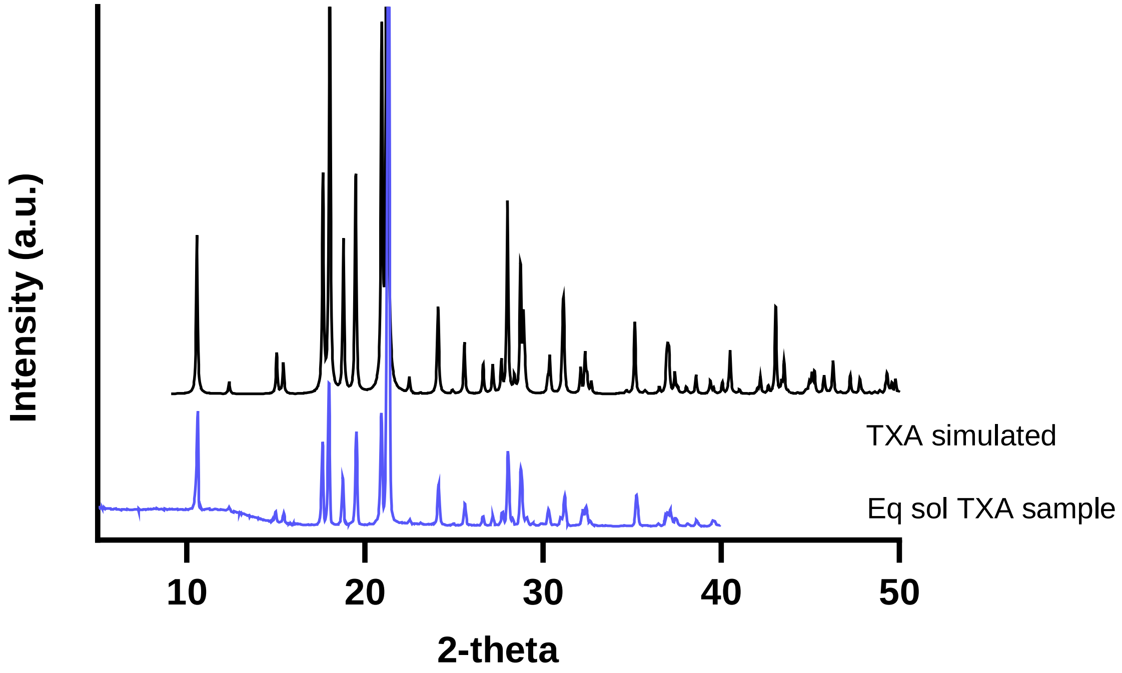

Figure S4. Overlay of powder X-ray diffraction patterns of **TXA** and solid residue obtained after equilibrium solubility studies of **TXA**.

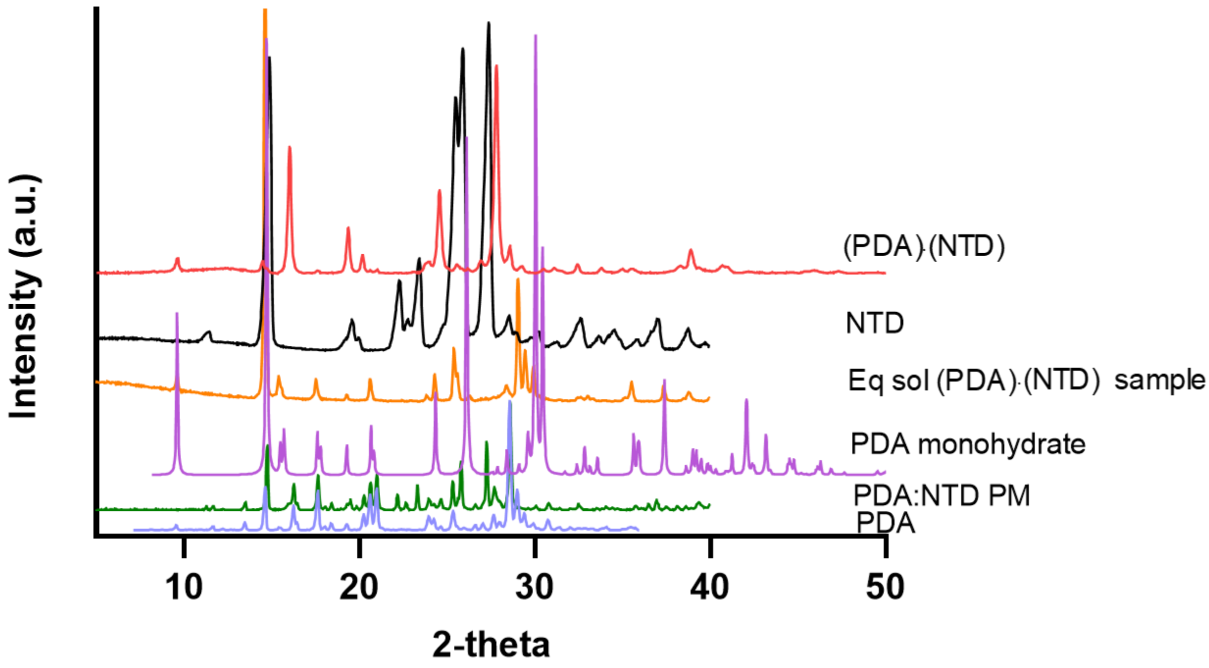

Figure S5. Overlays of powder X-ray diffraction patterns for **PDA**, **PDA** monohydrate, **NTD**, **PDA:NTD** PM, **(PDA)·(NTD)** and solid residue obtained after equilibrium solubility studies of **(PDA)·(NTD).**

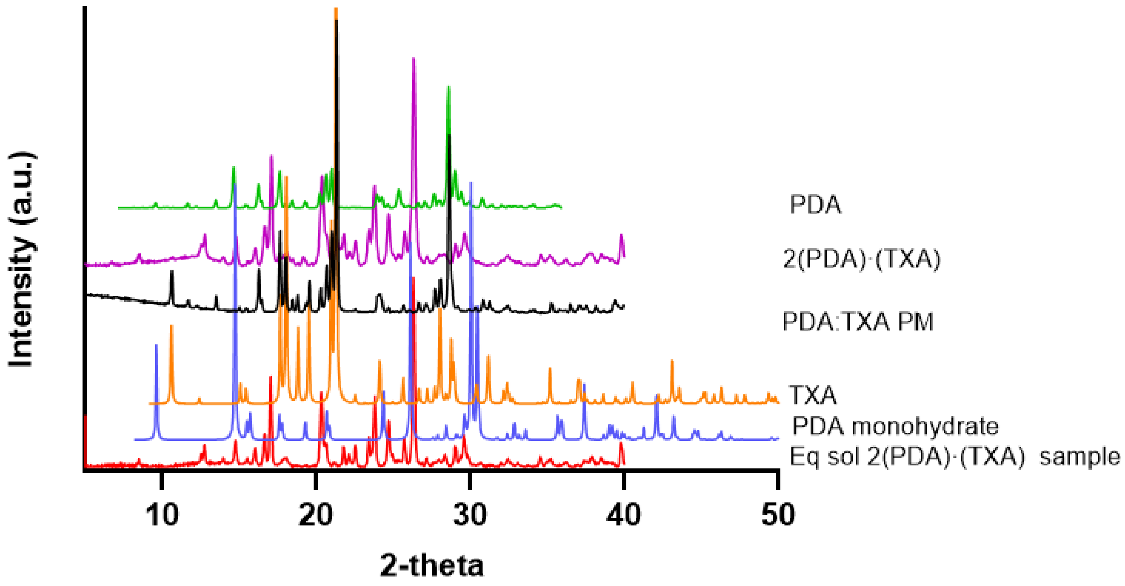

Figure S6. Overlay of powder X-ray diffraction patterns of **PDA**, **PDA** monohydrate, TXA, **2(PDA)·(TXA**) and solid residue obtained after equilibrium solubility studies of **2(PDA)·(TXA)**.

S4. Nuclear Magnetic Resonance (NMR) Spectroscopy Data

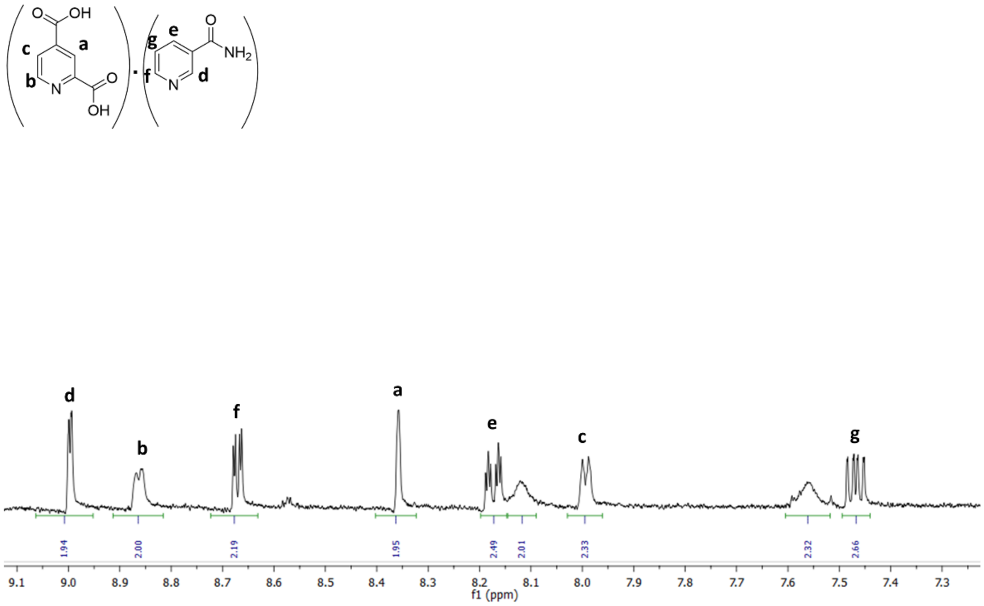

Figure S7. ^{1}H NMR (400 MHz, DMSO-d_6) spectrum of (**PDA**)·(**NTD**).

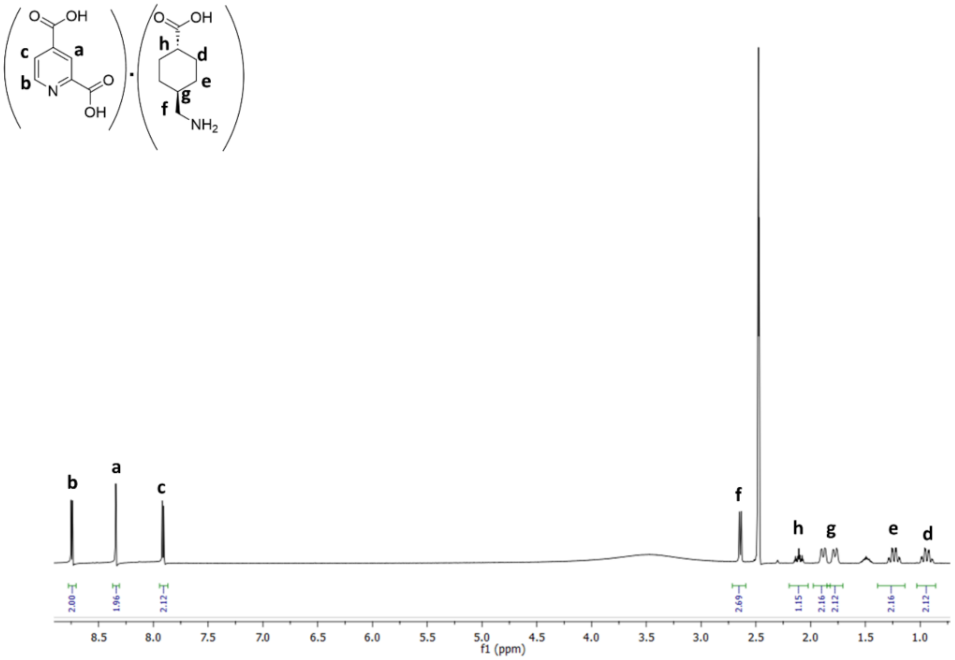

Figure S8. ^{1}H NMR (400 MHz, DMSO-d_6) spectrum of **2**(**PDA**)·(**TXA**).

S5. UV-Vis and HPLC Data

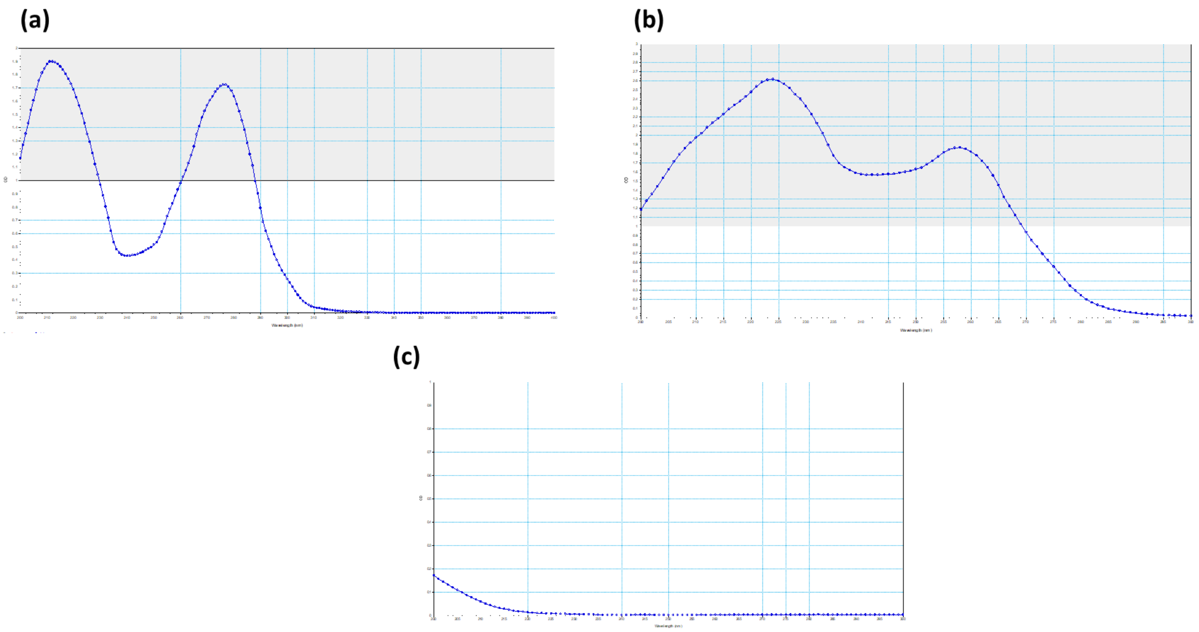

Figure S9. UV-spectra of (a) **PDA**, (b) **NTD**, (c) **TXA** recorded in range of 200-300 nm in methanol.

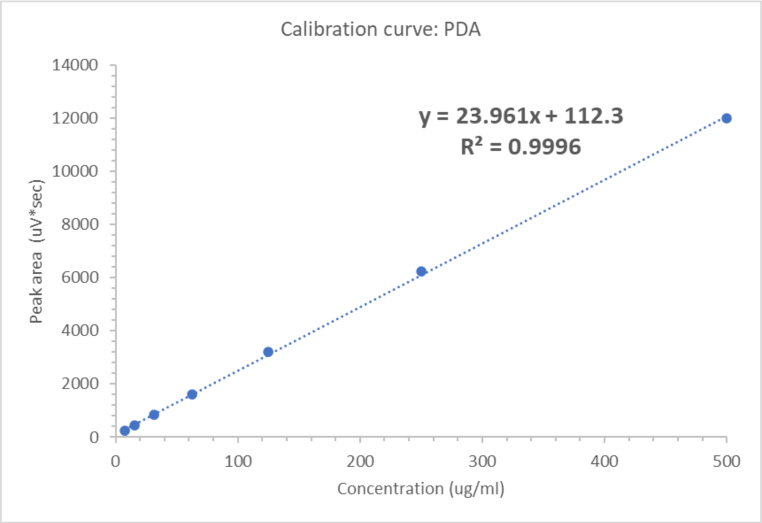

Figure S10. Calibration curve of **PDA** in range of 7.81 – 500 µg/ml for HPLC analysis.

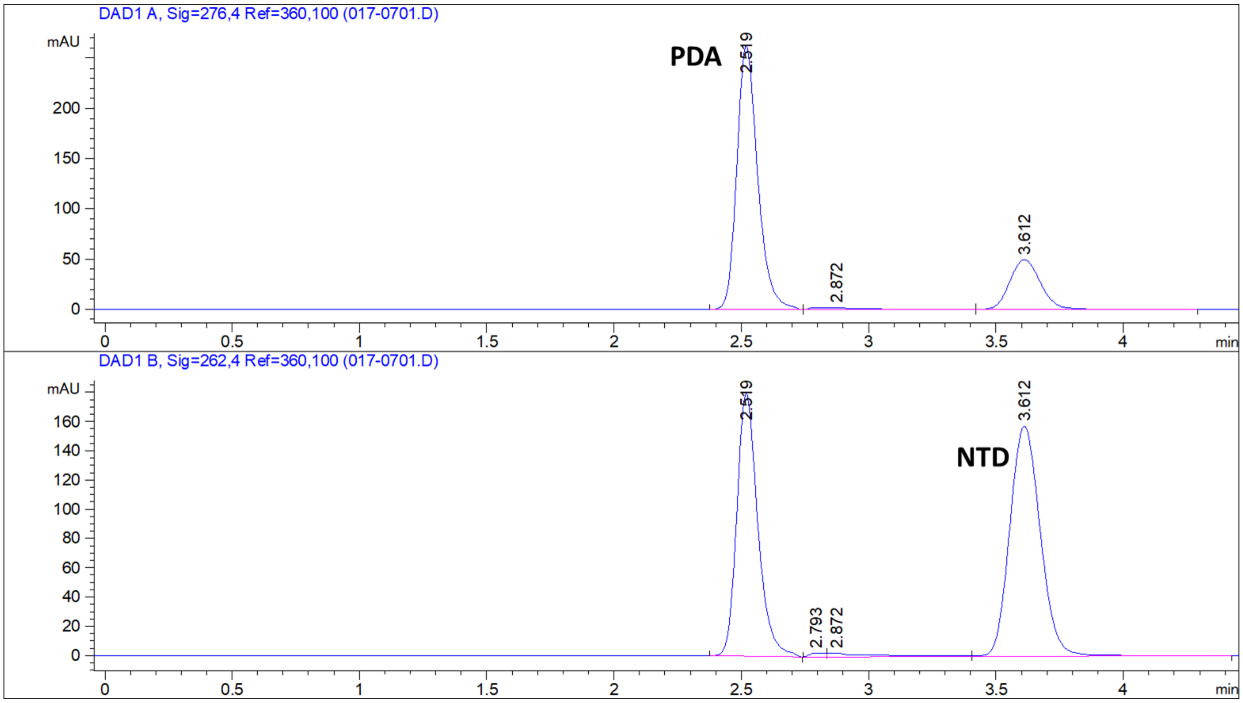

Figure S11. Chromatograms of **PDA** and **NTD** samples recorded at 262 and 276 nm showing well separated peaks of **PDA** and **NTD**.

S5. References

(1) Krause, L.; Herbst-Irmer, R.; Sheldrick, G. M.; Stalke, D. Comparison of Silver and Molybdenum Microfocus X-Ray Sources for Single-Crystal Structure Determination. *J. Appl. Crystallogr.* **2015**, *48* (1), 3–10. https://doi.org/10.1107/S1600576714022985.

(2) Dolomanov, O. V.; Bourhis, L. J.; Gildea, R. J.; Howard, J. A. K.; Puschmann, H. OLEX2: A Complete Structure Solution, Refinement and Analysis Program. *J. Appl. Crystallogr.* **2009**, *42* (2), 339–341. https://doi.org/10.1107/S0021889808042726.

(3) Sheldrick, G. M. Crystal Structure Refinement with SHELXL. *Acta Crystallogr. Sect. C Struct. Chem.* **2015**, *71* (Md), 3–8. https://doi.org/10.1107/S2053229614024218.
